# Supplementary material for: Highly Effective Proton-Conduction Matrix-Mixed Membrane Derived from an -SO3H Functionalized Polyamide
Source: Molecules. 2022 Jun 26;27(13):4110. doi: 10.3390/molecules27134110 (PMC9268481; doi:10.3390/molecules27134110)
Supplement: Supplementary file 1 [file molecules-27-04110-s001.zip › molecules-1753653-supplementary.pdf]

molecules

MDPI

Article

Highly Effective Proton-Conduction Matrix-Mixed Membrane Derived from an -SO_3H Functionalized Polyamide

Jamal Afzal [1], Yaomei Fu [2], Tian-Xiang Luan [1], Zhongmin Su [2,3] and Pei-Zhou Li [1,4,*]

1 School of Chemistry and Chemical Engineering, Shandong University, No.27 Shanda South Road, Jinan 250100, China; jamalsarwari46@gmail.com (J.A.); txluan@mail.sdu.edu.cn (T.-X.L.)
2 Shandong Peninsula Engineering Research Center of Comprehensive Brine Utilization, Weifang University of Science and Technology, Shouguang 262700, China; fuyaomei@wfust.edu.cn (Y.F.); zmsu@nenu.edu.cn (Z.S.)
3 School of Chemistry and Environmental Engineering, Changchun University of Science and Technology, Changchun 130022, China
4 Science Center for Material Creation and Energy Conversion, Institute of Frontier and Interdisciplinary Science, Shandong University, Qingdao 266237, China
* Correspondence: pzli@sdu.edu.cn

Experimental Section

General Characterizations. Fourier-transform infrared spectroscopy (FTIR) spectra were produced using a Bruker Optics instrument (Tensor II) with KBr pellets. Powder X-ray diffraction (PXRD) analyses were performed and PXRD patterns were recorded on a X-ray diffractometer (Rigaku Smart lab 9KW, Rigaku, Austin, TX, USA) using monochromatic Cu Kα radiation (1.5406 Å) measuring in the 2θ range from 5 to 70 ° with a 2 degree·min^{-1} scanning speed. N_2 adsorption–desorption isotherm measurements were performed on a Micromeritics ASAP 2020 system at 77 K. Before the sorption measurements, the samples were degassed for 12 h at 120 °C under vacuum. Highly pure (99.999%) gases were used in the measurements. Thermogravimetric analyses (TGA) were carried out by a Mettler Toledo TGA/SDTA851 machine (Mettler Toledo, Greifensee, Switzerland) under an N_2 atmosphere (99.999%) with a flow rate of 50 mL·min^{-1} and an operating temperature ranging from 25 to 800 ºC with a heating rate of 5 °C·min^{-1}. Scanning electron microscopy (SEM) images were produced using a JSM-7001F scanning electron microscope, which operated at 3.0 KV accelerating voltage. Transmission electron microscopy (TEM) images were produced using a JEOL JEM-1011 transmission electron microscope, operating at 200 KV. The data for the proton conductivities of materials were produced using CH Instruments (CHI760E) using the quasi-four-probe method and an impedance/gain-phase analyzer in a 1–10 MHz frequency range and a 200 mV AC amplitude.

Proton conductivity measurements of Pure Samples. AC impedance data and electrical properties of the materials were measured at a wide range of temperatures (303–353K) and humidity conditions (75%–98%). For electrical measurements, the powder samples were firstly ground homogeneously in mortars for 10 minutes, and then cash-like discs a diameter of 6 mm and thickness ranging from 2 to 4.5 mm were prepared by compressing the materials under a pressure of 7 MPa for 30 s using a tablet press. Copper-based electrodes and home-made sample holders were utilized, in which the prepared sample discs were placed for the measurement of resistivity and proton conductivity. The sample discs with electrodes were then placed in temperature- and humidity-controlled chambers. Before measurement, the sample discs with electrodes were placed in a chamber with a specific temperature and relative humidity for 48 h to balance the inside of the sample discs with the setting conditions. Further, to improve the accuracy of the results, the impedance measurements were repeatedly collected until convergent and uniform results were obtained, and the Nyquist plot was evaluated. Proton conductivity

measurements were carried out in the frequency range of 1–10^6 Hz, 100 mV amplitude, and parallel electrode assembly was used to produce the Nyquist plot. The equivalent circuit was designed, by which the resistance was acquired, and the impedance plot was fitted. To analyze this, Z view software (version 3.5) was used. Capacitance and resistance were placed parallel to each other in designing the equivalent circuit and measuring resistance.

The proton conductivity of the sample discs was calculated though the formula:

$$\sigma = \frac{l}{RS}$$

where l and S represent the disc's thickness (cm) and cross-sectional area (cm^2), respectively. In a Nyquist plot utilizing Z view software, R is the disc's bulk resistance, which is fitted by the equivalent circuit of the semicircle.

Their activation energies during proton conduction processes were calculated by the formula:

$$\ln(\sigma T) = \ln A - E_a/k \times 1/T$$

where T is the absolute temperature in kelvin, E_a is the activation energy, and k is the Boltzmann constant.

Physicochemical Investigations of matrix-mixed Membranes

Ion exchange capacity (IEC). The ion exchange capacity (IEC) of matrix-mixed membranes was measured using an acid–base titration method. For one day, dried pre-weighted membranes were immersed in a 2 M NaCl solution to replace proton via sodium ions, which were then followed by the liberated positive ions. To determine the endpoint, phenolphthalein was used as an indicator, and the solution was titrated with 0.01 M NaOH solution. The given equation was used to calculate IEC values. [1,2]

$$\text{IEC (mmol/g)} = \frac{C_{NaOH} \cdot V_{NaOH}}{W_{dry}} \quad \text{(S1)}$$

where, C_{NaOH} is the concentration of NaOH solution (mol/g) and V_{NaOH} is the volume of consumed NaOH solution (mL), respectively.

Water Uptake property and Swelling characteristics. To study the water uptake property, the membranes was dried at 80 °C for 6 h and its weight was measured. The membranes were then immersed in distilled H_2O at 30 and 80 °C overnight, removed from the H_2O, and rapidly dried using clean paper to determine the weight of the membranes once more. The following Equation (S2) was used to calculate the water uptake (%) of matrix-mixed membranes [3,4].

$$\text{WU(\%)} = \frac{M_{wet} - M_{dry}}{M_{dry}} \times 100 \quad \text{(S2)}$$

where M_{wet} and M_{dry} are the weights of wet and dry sample respectively.

To study the swelling property, the membranes was dried at 80 °C for 6 h and its weight was measured. The membranes were then immersed in distilled H_2O at 30 °C and 80 °C overnight, removed from the H_2O, and rapidly dried using clean paper to determine the weight of the membranes once more.[3] The Equation (S3) was used to calculate the swelling ratio (%) of matrix-mixed membranes.

$$\text{Swelling ratio } (\Delta L)\ (\%) = \frac{L_{wet} - L_{dry}}{L_{dry}} \times 100 \quad \text{(S3)}$$

where L wet and L_{dry} are the diameters of wet and dry membranes respectively in round form.

Dimensional Stability. To investigate the dimensional stability of the matrix-mixed membrane, the membranes were submerged in distilled water for 5 h at 30 °C and 80 °C respectively. The membranes' length and width were measured with a vernier caliper. The membrane was then dried and the change in length and width was measured at room

temperature. The change in length (ΔL_c) of the membrane was calculated by using the Equation (S4). [3,4]

$$(\Delta L_c)\ (\%) = \frac{L - L_{dry}}{L_{dry}} \times 100 \quad \text{(S4)}$$

where L and L_{dry} are the length of wet and dry membranes respectively.

The change in weight (ΔW_c) of the membrane was calculated by using the Equation (S5).

$$(\Delta W_c)\ (\%) = \frac{W - W_{dry}}{W_{dry}} \times 100 \quad \text{(S5)}$$

where W and W_{dry} are the width of wet and dry membranes respectively.

Chemical Stability. A small piece of the matrix-mixed membrane PA-$PhSO_3H$-MMM with different ratios was immersed in Fenton reagent ($FeSO_4$ in H_2O_2 (3%), 3.0 ppm) at 30 °C and 80 °C to investigate its chemical stability. The oxidative stability was determined by the elapsed time (t) it took for the membrane to dissolve completely. The results show that at 80 °C, all the membranes have an elapsed time (t) value of ≥6 h. This is the standard test to measure the chemical stability of matrix-mixed membranes and the results indicate that these membranes have good chemical stability due to the aromatic structure of sulfonated polyamides. [3]

Hydrolytic Stability. To check the hydrolytic stability of the matrix-mixed membranes towards water, the membranes were immersed in distilled water at 50 °C and measuring the elapsed time until the hydrated membranes lost their mechanical properties. When the membranes were slightly bent, they broke, which was the criterion for loss of mechanical property. [2]

Proton conductivity measurements of matrix-mixed membrane. The fabricated membranes were cut into rectangles for the proton conduction measurements.

It is calculated by using the following equation:

$$\sigma = \frac{l}{WRT}$$

where l is the length, R is the measured resistance, T is thickness of membrane, and W is the width of membrane.

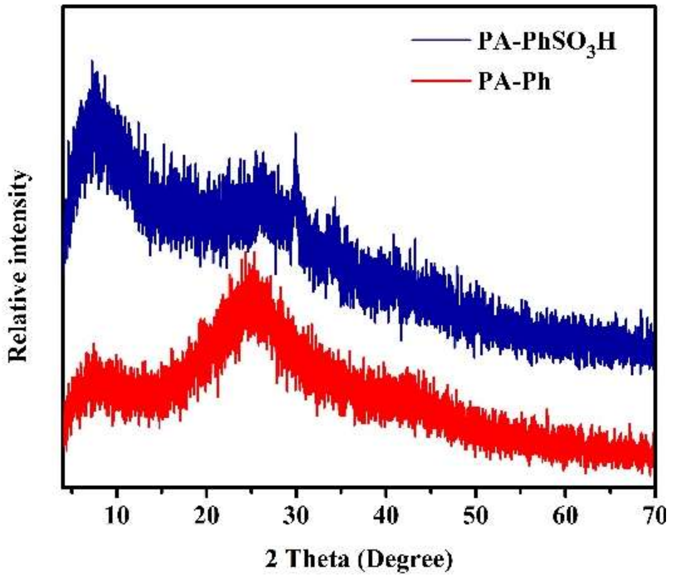

Figure S1. PXRD patterns of PA-$PhSO_3H$ and PA-Ph.

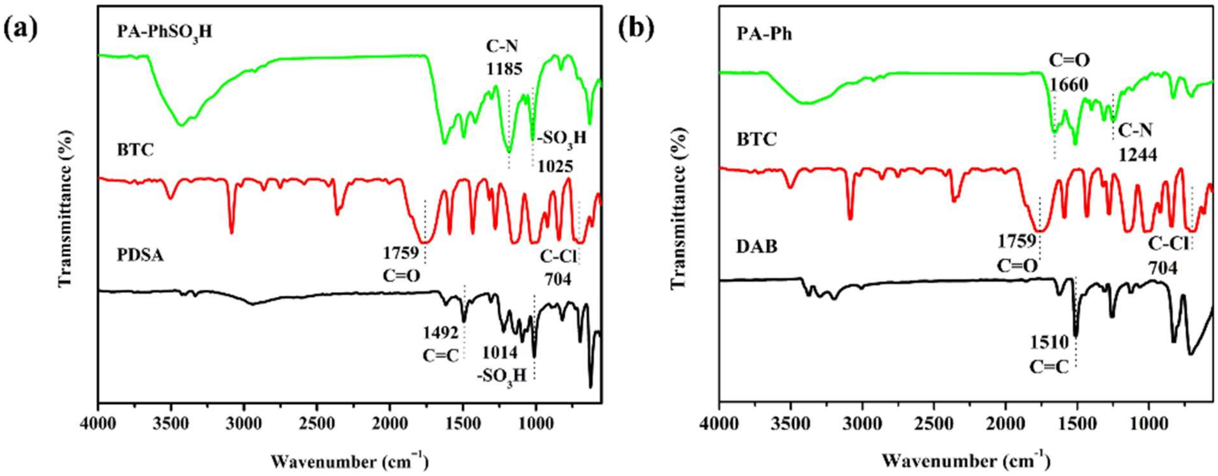

Figure S2. FTIR spectra of (a) **PA-PhSO₃H** and (b) **PA-Ph** and the related reactants.

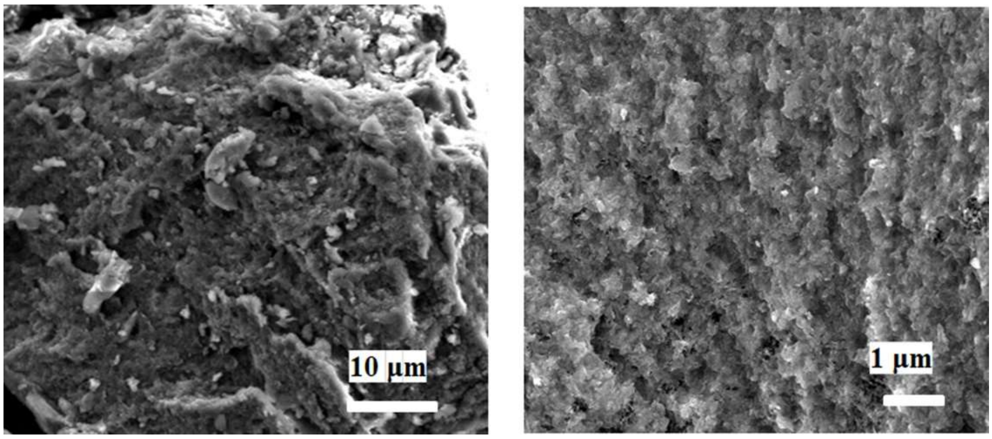

Figure S3. (**a**) SEM images of **PA-PhSO₃H**.

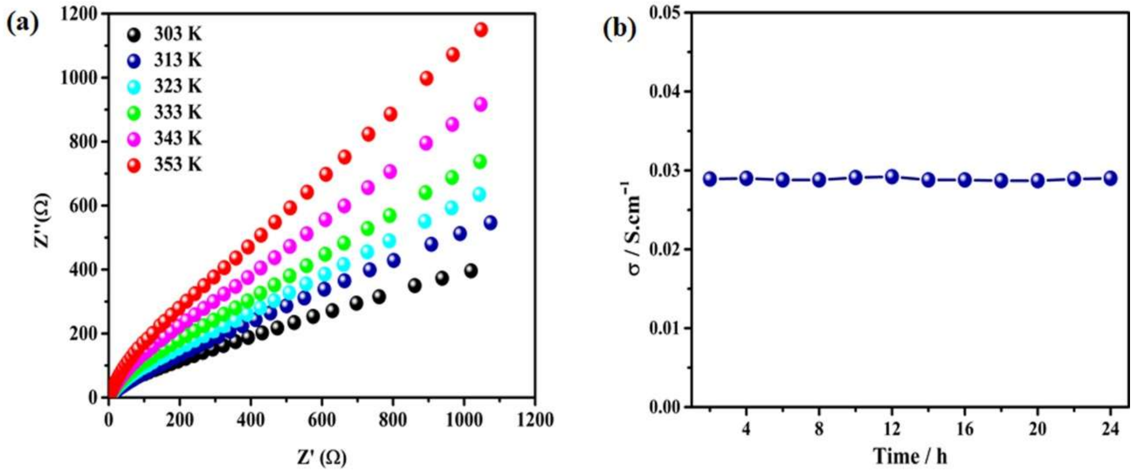

Figure S4. Nyquist plot and Long-life reusability of **PA-PhSO3H-MMM (1:1)** at 98%RH and different temperatures.

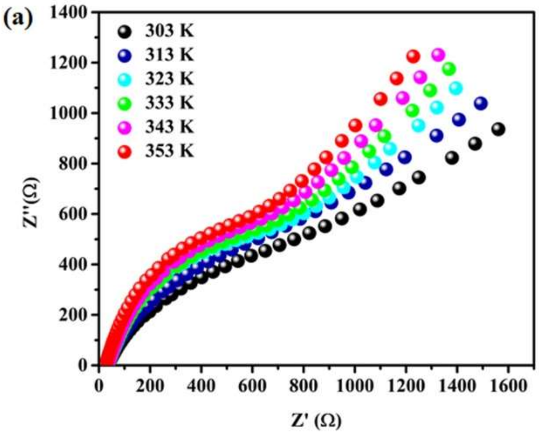

Figure S5. Nyquist plot of **PA-PhSO3H-MMM (0.4:1)** at 98%RH and different temperatures.

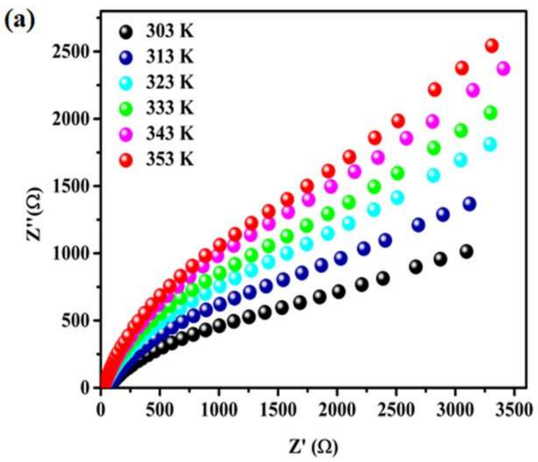

Figure S6. Nyquist plot of **PA-PhSO3H-MMM (0.1:1)** at 98%RH and different temperatures.

References

1. Khan, M. I.; Khraisheh, M.; AlMomani, F., Innovative BPPO Anion Exchange Membranes Formulation Using Diffusion Dialysis-Enhanced Acid Regeneration System. *Membranes* **2021,** *11* (5), 311. [https://doi.org/10.3390/membranes11050311]

2. Ali, F.; Saeed, S.; Shah, S. S.; Rahim, F.; Duclaux, L.; Levêque, J.-M.; Reinert, L., Sulfonated Polyimide-Clay Thin Films for Energy Application. *Recent Patents on Nanotechnology* **2016,** *10* (3), 221-230. [https://doi.org/10.2174/1872210510666160429144718]

3. Wang, C.; Shen, B.; Zhou, Y.; Xu, C.; Chen, W.; Zhao, X.; Li, J., Sulfonated aromatic polyamides containing nitrile groups as proton exchange fuel cell membranes. *Int J Hydrogen Energy* **2015,** *40 (19)*, 6422-6429. [https://doi.org/10.1016/j.ijhydene.2015.03.078]

4. Wang, C.; Li, N.; Shin, D. W.; Lee, S. Y.; Kang, N. R.; Lee, Y. M.; Guiver, M. D., Fluorene-Based Poly(arylene ether sulfone)s Containing Clustered Flexible Pendant Sulfonic Acids as Proton Exchange Membranes. *Macromolecules* **2011,** *44* (18), 7296-7306. [https://doi.org/10.1021/ma2015968]
